# Land Use Influences Niche Size and the Assimilation of Resources by Benthic Macroinvertebrates in Tropical Headwater Streams

**DOI:** 10.1371/journal.pone.0150527

**Published:** 2016-03-02

**Authors:** Diego Marcel Parreira de Castro, Débora Reis de Carvalho, Paulo dos Santos Pompeu, Marcelo Zacharias Moreira, Gabriela Bielefeld Nardoto, Marcos Callisto

**Affiliations:** 1 Universidade Federal de Minas Gerais, Instituto de Ciências Biológicas, Departamento de Biologia Geral, Laboratório de Ecologia de Bentos, Belo Horizonte, MG, Brasil; 2 Universidade Federal de Lavras, Departamento de Biologia, Setor de Ecologia, Laboratório de Ecologia de Peixes, Lavras, MG, Brasil; 3 Universidade de São Paulo, Centro de Energia Nuclear na Agricultura–CENA, Laboratório de Ecologia Isotópica, Piracicaba, SP, Brasil; 4 Universidade de Brasília, Instituto de Ciências Biológicas, Departamento de Ecologia, Brasília, DF, Brasil; University of Brighton, UNITED KINGDOM

## Abstract

It is well recognized that assemblage structure of stream macroinvertebrates changes with alterations in catchment or local land use. Our objective was to understand how the trophic ecology of benthic macroinvertebrate assemblages responds to land use changes in tropical streams. We used the isotope methodology to assess how energy flow and trophic relations among macroinvertebrates were affected in environments affected by different land uses (natural cover, pasture, sugar cane plantation). Macroinvertebrates were sampled and categorized into functional feeding groups, and available trophic resources were sampled and evaluated for the isotopic composition of ^13^C and ^15^N along streams located in the Cerrado (neotropical savanna). Streams altered by pasture or sugar cane had wider and more overlapped trophic niches, which corresponded to more generalist feeding habits. In contrast, trophic groups in streams with native vegetation had narrower trophic niches with smaller overlaps, suggesting greater specialization. Pasture sites had greater ranges of resources exploited, indicating higher trophic diversity than sites with natural cover and sugar cane plantation. We conclude that agricultural land uses appears to alter the food base and shift macroinvertebrate assemblages towards more generalist feeding behaviors and greater overlap of the trophic niches.

## Introduction

Tropical streams are among the most threatened ecosystems in the world [1], especially in developing countries [2]. In recent decades, these environments have been experiencing substantial changes in land use and occupation. Such changes include replacing native vegetation with large-scale agricultural activities and poorly planned urban expansion.

Those changes in turn have resulted in alarming losses of biodiversity in aquatic ecosystems, especially in tropical streams [1,3]. The Cerrado (neotropical savanna) is the second largest biome in South America (after the Amazon), a biodiversity hotspot [4] and one of the most threatened biomes in the world, mainly because of the replacement of natural vegetation with pasture and row crop agriculture [5,6]. Those agricultural activities currently alter 40% of the native terrestrial plant cover [7] and have reduced or removed the native riparian vegetation, thereby degrading aquatic ecosystem ecological integrity [8].

The replacement, reduction or removal of vegetation cover, especially in riparian areas, leads to the degradation of physical habitat structure, increased sedimentation rates, hydrological changes, and water temperature oscillations [9,10]. These changes directly influence the input of nutrients, allochthonous resources, autochthonous production [10], the quantity and quality of available food resources [9], and may simplify trophic structure and reduce biological diversity [11,12]. Therefore, studies addressing the impacts of changes in vegetation cover on the energy flow and trophic relations in aquatic environments are essential to an understanding of the mechanisms that regulate the ecological integrity of those environments.

In freshwater ecosystems, aquatic invertebrates are the main link between primary producers (e.g., periphyton and aquatic macrophytes) and higher trophic levels (e.g., aquatic vertebrates). By breaking down organic matter, they contribute to litter decomposition and nutrient availability for other organisms [13,14]. Aquatic macroinvertebrates may be classified according to their feeding habits into functional feeding groups (FFG) [15] based on morphological and behavioral characteristics [16]. Those groups include the following: (i) scrapers that feed on organic matter adhered to organic and inorganic substrates (e.g., periphyton, algae and their associated microbiota); (ii) shredders that feed directly on coarse particulate organic matter (CPOM); (iii) gathering-collectors that feed mainly on deposited fine particulate organic matter (FPOM); (iv) filtering-collectors that filter fine suspended organic matter; and (v) predators that feed on whole animals or their parts [14,16].

Macroinvertebrate assemblages are sensitive to environmental conditions and reflect the physical and chemical conditions of the ecosystem [17]. Therefore, analyzing trophic relationships among macroinvertebrates and the energy flow in aquatic ecosystems is required to understand assemblage structure and dynamics and ecosystem functioning [18].

The energy flow and trophic relationships among the organisms in an ecosystem may be assessed using stable isotope analysis (SIA) of carbon (C) and nitrogen (N) [19]. The ratios between stable isotopes of ^13^C and ^12^C (expressed relative to a standard and called *δ*^13^C) and of ^15^N and ^14^N (expressed relative to a standard and called *δ*^15^N) provide information that incorporates spatio-temporal scales and facilitates the analysis of food assimilation by consumers [20] and the definition of their trophic niches [21,22]. Stable isotope analysis has been an important and advantageous tool in trophic ecology studies [23] to examine resource partitioning [24], ecosystem fluxes of carbon and nitrogen [25], to reconstruct diets [26,27] and to characterize niche properties [22,28].

The *δ*^13^C and *δ*^15^N values in consumers reflect the C and N stable isotope ratios of the food sources [20]. The ^13^C enrichment between food sources and consumers is usually low (0–1‰) [29,30]. Because *δ*^13^C values typically differ among basal sources (e.g., plant material from C3 and C4 plants), *δ*^13^C is used as an indicator of C sources for certain consumers along food chains [18,20]. In contrast, the trophic fractionation of *δ*^15^N usually varies from 2 to 4‰ at each trophic level [29,30], facilitating definition of the total length of the food chain and the position of an organism within it [18,31]. Therefore, the isotopic ratios of *δ*^13^C and *δ*^15^N in animal tissues reflect information on their use of physical habitats and trophic characteristics and are currently used to determine organic matter origin, trophic relationships and niche size and overlap [22,28].

The relative contributions of food resources to the diet of an animal may be calculated using isotope mixing models [32]. However, most of food webs are too complex and the number of food sources exceeds the number of useful isotope tracers by more than one. In this case, the model does not generate exact values for proportional contributions of each source, but instead provides a range of possible contributions or feasible solutions [19]. Recently Bayesian mixing models have been proposed to assess stable isotope data (e.g., [33–36]) through the use of statistical distributions to characterize the uncertainties in food sources, consumer isotopic values, and estimated source contributions [19].

The isotopic C and N signatures of consumers in aquatic ecosystems may vary because of changes in riparian zones, which provide most of the organic matter used by aquatic communities [27]. In addition, riparian vegetation stabilizes stream banks and filters excessive inputs of materials (e.g., fine sediments) and nutrients (e.g., manure and fertilizers used in surrounding plantations) to the waterbodies [9,37]. Therefore, variations in riparian vegetation cover influence the dynamics and structure of aquatic communities (e.g. [38,39]), changing the isotopic composition of resources and consumers. In turn, those isotopic signatures aid comparisons of the ecological processes in riparian zones, identification of the effects of agriculture and deforestation on assemblages, and assessments of the interactions between riparian land cover and water bodies [20,40].

In this study, we evaluated how the energy flow and the trophic relationships among benthic macroinvertebrates were influenced by riparian land uses (natural vegetation, pasture, sugar cane plantation). Based on C and N stable isotope analyses, we compared isotopic niche breadth and the degree of niche overlap among macroinvertebrate trophic groups. We first tested whether anthropogenic activities in areas adjacent to streams can expand the trophic niche of macroinvertebrates and their degree of overlap. Then, we assessed whether anthropogenic land uses in riparian zones were associated with more generalist trophic groups and less specialized trophic groups compared with streams with native riparian vegetation.

## Materials and Methods

### Study area

**Fig 1:**
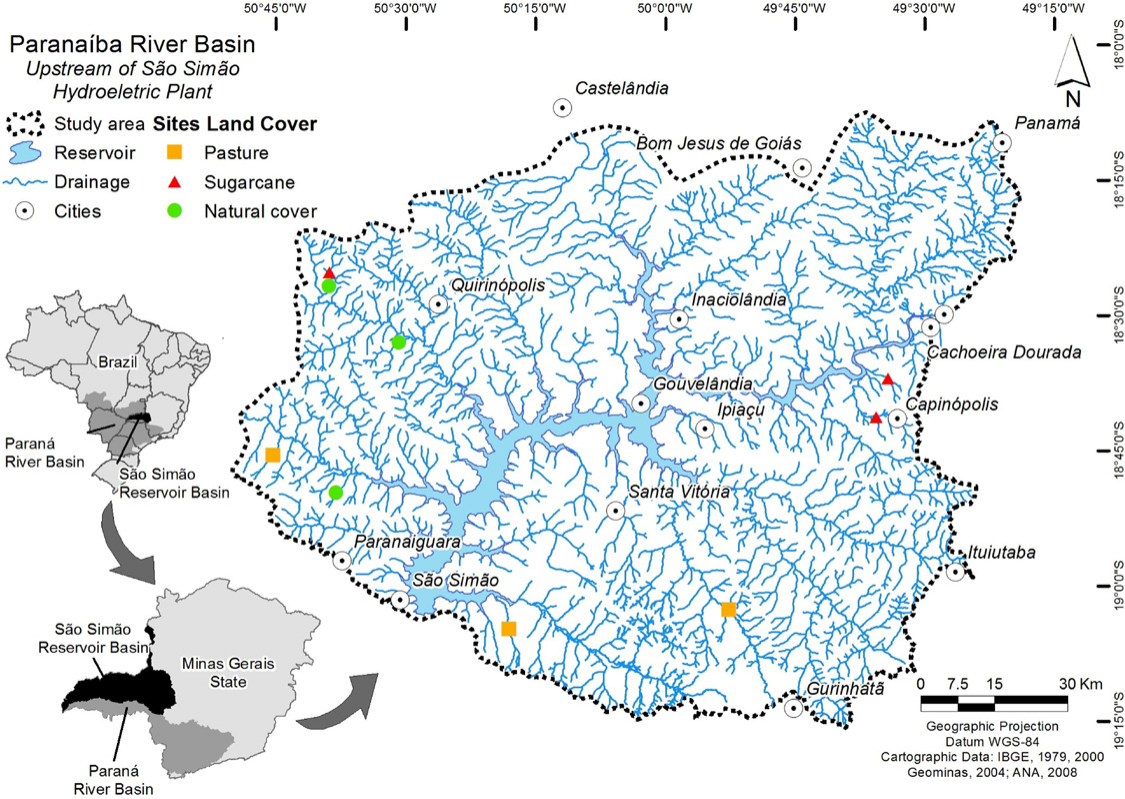
Locations of the nine stream sites selected according to their land use and study area in the states of Minas Gerais and Goiás, Brazil.

We studied sites in tributaries of the São Simão Hydroelectric Power Plant Reservoir, located in the sub-basin of the Paranaíba River, southeastern Brazil (Fig 1). The Paranaíba River basin is the second largest drainage basin of the Paraná River basin, corresponding to a drainage area of 223 km^2^ (25.4% of its area) [27].

Nine 2^nd^- and 3^rd^-order streams (on a 1:100,000 scale map) located in the states of Minas Gerais and Goiás were selected from 110 previously investigated streams [41]. We used a hierarchical and spatially balanced sampling algorithm (e.g., [8]) proposed by Olsen & Peck [42] to select those 110 sampling sites. The nine streams were selected according to their land use, where three were located in pasture areas, three were located in sugar cane plantation areas, and three were representative of natural riparian vegetation. A continuous segment proportional to the width of the stream (defined as 40 times the mean stream width, minimum of 150 meters) was sampled in each stream. We sampled available trophic resources and aquatic macroinvertebrates during the dry season (September) of 2012. We evaluated the land use of the riparian zones of the sites through use of satellite images [43]. For the nine sites, we determined the percentages of natural cover, pasture, and sugar cane plantations in a 150 m radius buffer around the upstream limit of each site [27]. To illustrate the variation in the physical habitat structure of streams with different land uses, environmental characteristics of each site were quantified (S1 Table) and are detailed in Carvalho et al. [27].

### Sample collection and processing

Each stream was subdivided into five equally sections. We collected five independent samples (one per section) of benthic macroinvertebrates from each site, along with five independent samples of food resources: CPOM, FPOM, filamentous algae, periphyton, leaves of the riparian vegetation (forest, pasture, sugar cane) and suspended particulate organic matter (seston). Only one site had aquatic macrophytes; therefore, macrophytes were not considered as resources in the analyses. We collected algae, CPOM, periphyton, FPOM, leaves of native riparian vegetation and seston from sites in all three types of riparian environments, whereas pasture grasses were collected only in the pasture environment and sugar cane leaves only in the sugar cane environments (S2 Table).

We collected benthic macroinvertebrates assemblages through use of D-frame kick-nets (30 cm aperture, 500 mm mesh), following a systematic zig-zag pattern along the segments defined [44], covering all different substrates and habitats in each site. Five sample units (0.09 m^2^ each) were taken per stream, one per section, totaling 0.45 m^2^ per site. All invertebrates sampled were stored on ice and after 2 days processed in laboratory. In the laboratory, the organisms collected were washed in distilled water, taxonomically identified [16,45,46] and classified into functional feeding groups: predators, scrapers, shredders, gathering-collectors (hereafter “collectors”) and filtering-collectors (hereafter “filter-feeders”) [47–49]. Shredders were divided into insect-shredders (Insecta) and shrimp-shredders (Crustacea) because shrimp-shredders may have a generalist omnivore behavior, feeding on multiple resources [50]. Each macroinvertebrate functional feeding group was considered a consumer, whereas the periphyton, filamentous algae, seston, FPOM, CPOM and vegetation leaves were considered resources. The other macroinvertebrate functional feeding groups were considered as resources for predators. It was not always possible to obtain five samples of each functional feeding group or food resources in each site (e.g. just three samples of insect-shredders in the pasture areas), therefore, a total 232 food resource samples and 202 macroinvertebrate samples (among 270 possible: 9 sites x 5 samples x 6 resources / FFG) were obtained and analyzed. The last author, MC, has a permanent license to collect aquatic invertebrates (10365–2) in the entire Brazilian territory provided by IBAMA/Sisbio, in accordance with federal law and the regulations of the Brazilian Environmental Ministry. The sampling sites were private, and permission from the owner or manager was obtained prior to sampling. None of the sampled species was protected by Brazilian law or red-listed.

After identifying and classifying in functional feeding groups, organisms were then oven-dried at 60° for 48 h, ground to a fine and homogeneous powder using a mortar and pestle and then stored in Eppendorf tubes for subsequent analysis of their isotopic compositions. Collector, filter-feeder, predator and scraper consumers were found at sites in all three types of riparian environments, whereas shrimp-shredders were not found in the sugar cane sites and insect-shredders were not found in natural cover sites. Each FFG sample was composed of only one specific family and different numbers of invertebrates were used for each sample to reach a minimal amount of material for isotope analysis (Table 1).

**Table 1:** Taxa used in each trophic group analyzed. The letter “n” indicates the number of samples. Different numbers of invertebrates were used for each sample to reach a minimal amount of material for isotope analysis.

Consumers	Natural cover	(n)	Pasture	(n)	Sugar cane	(n)
**Collectors**	Chironomidae	4	Baetidae	2	Chironomidae	4
	Elmidae (larvae)	9	Chironomidae	3	Elmidae (larvae)	10
	Leptohyphidae	2	Elmidae (larvae)	6	Leptohyphidae	1
			Leptohyphidae	1		
**Filter-feeders**	Hydropsychidae	11	Hydropsychidae	11	Hydropsychidae	11
	Philopotamidae	3	Leptoceridae	3	Simuliidae	4
	Simuliidae	1	Simuliidae	1		
**Shrimp-shredders**	Palaemonidae	13	Palaemonidae	7	−	
**Insect-shredders**			Calamoceratidae	2	Calamoceratidae	1
	−		Odontoceridae	1	Odontoceridae	1
			Pyralidae	3	Pyralidae	1
**Scrapers**	Elmidae (adult)	6	Ampullariidae	2	Elmidae (adult)	6
	Leptophlebiidae	7	Elmidae (adult)	7	Leptophlebiidae	4
	Psephenidae	2	Leptophlebiidae	3	Planorbidae	4
**Predators**	Megaloptera	6	Megaloptera	3	Belastomatidae	1
	Naucoridae	2	Naucoridae	3	Megaloptera	3
	Odonata	6	Odonata	8	Naucoridae	2
	Perlidae	1	Perlidae	1	Odonata	8
					Perlidae	1

Sampling of resources was carried out in parallel to macroinvertebrates collecting along the segments defined in each site. Periphyton was collected by scraping rocks with a brush (three rocks per segment) and placing the material in a plastic container with distilled water [44]. Seston was collected with a phytoplankton net (0.45 mm) set for 1 min upstream of each site. The samples were stored in coolers with ice after sampling and then transported to the laboratory, where they were kept frozen until processing. In the laboratory, the samples were filtered using a filtration apparatus coupled to a vacuum pump with calcined glass fiber filters (Millipore 45 *μm*). Filamentous algae was collected manually in each segment, stored in plastic containers in ice coolers and then frozen. The FPOM samples were collected from sediment deposits revolving the sediment and passing a phytoplankton net (0.45 mm) in the material in suspension. After the material was stored in plastic containers and then frozen. Pasture leaves, sugar cane leaves, and leaves of the natural riparian vegetation were manually collected along the segments delimited in each sampled stream, with the most common species being prioritized at the site. Species priorization was made in compliance with the most common and abundant species in each segment. Five leaves were then collected from each of the five most common plants. We obtained samples of native riparian vegetation even at sugar cane and pasture sites. The CPOM was randomly collected from leaf litter deposits in the streams. All leaves were then stored in paper bags and kept in plant presses until processing in the laboratory. In the laboratory, all resource samples were dried in an oven at 60°C for 48 h and then ground with a mortar and pestle and stored in Eppendorf tubes. Approximately 2–5 mg of dried animal tissue and 5–10 mg of resources were used for the isotopic analysis.

All samples were sent to the Laboratory of Isotope Ecology of the Center for Nuclear Energy in Agriculture (Centro de Energia Nuclear na Agricultura—CENA), University of São Paulo (Universidade de São Paulo), Piracicaba, Brazil, for determination of the *δ*^13^C and *δ*^15^N values. Analyses of isotopic ratios were processed through sample combustion under a continuous flow of ultrapure helium in an elemental analyzer (Carlo Erba, CHN– 1110) that was coupled to a Thermo Finnigan Delta Plus mass spectrometer for isotopic ratios. The results were expressed in delta notation (*δ*), in parts per thousand (‰), relative to standard international references (V-PDB–Vienna Pee Dee Belemnite for C and atmospheric air for N), and were calculated using the following equation:

$$\delta X=[(R_{Sample}/R_{Standard})-1]\times 10^{3}$$

where X is ^13^C or ^15^N and R represents the isotopic ratios ^13^C/^12^C or ^15^N/^14^N [25]. The analytical precision values estimated by replicates of the working standards of *δ*^13^C and *δ*^15^N were ±0.10 and ±0.11‰, respectively.

When using stable isotope data to reconstruct animal diets, the resources must have isotopically distinct signatures to ensure a sensitive interpretation of the results. If they are not significantly different and are somehow logically related (e.g., same taxon or trophic group), the resources may be combined and represented in the mixing model by a single set of isotopic values [51]. The *δ*^13^C values of samples of the riparian vegetation and CPOM deposited in the streams were very similar among the environments; therefore, the values of the riparian vegetation samples were excluded from the analyses. The FPOM and the seston samples also had similar *δ*^13^C values and were grouped; hereafter, those groups are called fine particulate organic matter (FPOM).

### Data analysis

We used the SIAR package for the analysis of stable isotopes [34,52] in R [53], to determine the relative contribution of each food resource available for the macroinvertebrates. Differences in the isotopic ratios of the food resources and consumers among environments were tested using one-way analyses of variance (ANOVAs) when the normality and homoscedasticity assumptions were met. The nonparametric Kruskal-Wallis test was used for data with non-normal distribution.

In the partition analysis, the food resources of each land use category were considered separately to determine the contribution of each resource for the consumers. The mean value of the food resources in all categories was used to visually represent the spatial distribution of the taxa according to their *δ*^15^N and *δ*^13^C values. The fractionation values used were 0.5 ± 0.13‰ for C and 2.3 ± 0.18‰ for N [29]. The trophic structure of the benthic macroinvertebrate assemblage was described for each land use using the metrics proposed by Layman et al. [21]. Those metrics use the stable isotope ratios of the different components of the food chain to describe the niche and trophic structure of the assemblage, providing information on the trophic diversity and redundancy within a food chain [54]. However, one of the limitations of using the metrics originally proposed by Layman et al. [21] is that those metrics are sensitive to the sample size and may not be comparable between studies and different sites. A second limitation is that the metrics, when applied to an assemblage, do not incorporate any natural variability within the system and, thus, provide only a point estimate of each metric [55].

A Bayesian approach recently developed for the aforementioned metrics enables the distribution of the sampling errors of the means estimated for the members of the assemblage. Using that approach, we generated a posterior distribution of the estimates of those metrics, providing a measure of uncertainty and allowing statistical comparisons among assemblages [54,55]. Thus, we calculated the five macroinvertebrate assemblage metrics through use of the Stable Isotope Bayesian Ellipses package in R (SIBER; [55]): 1) *δ*^13^C range (CR_b_) and *δ*^15^N range (NR_b_), which together indicate the variety of resources exploited by the assemblage. 2) The mean distance to centroid (CD_b_), which is the mean Euclidian distance of each assemblage component to the centroid, indicating the trophic diversity within the food chain. 3) The mean nearest neighbor distance (MNND_b_), which is the mean Euclidean distance from each group to its nearest neighbor in the *δ*^13^C-*δ*^15^N bi-plot space (plotted based on their mean stable isotope signatures), an estimate of the total density and clustering within the assemblage. Low MNND values indicate an increase in trophic redundancy, i.e., the occurrence of many groups with similar trophic levels. 4) The standard deviation of the nearest neighbor distance (SDNND), which measures the uniformity of the groups in bi-plot space, where lower SDNND values suggest a more uniform trophic niche distribution [21]. We calculated the metrics originally proposed by Layman et al. [21] and reformulated in a Bayesian framework by Jackson et al. [55] for each site, enabling a comparison of the structure and trophic ecology of each land use. Results were then compared between land uses based on the visual analysis of the credible intervals (CIs) of the Bayesian implementation of the Layman metrics. We estimated the standard ellipse area (SEA_c,_ in ‰^2^) as a bivariate measure of the central mean of the isotopic niche [55]. The SEA_c_ enables calculating the degree of niche overlap of the assemblage (in %, where 100% indicates total overlap) and may be used as a quantitative measure of diet similarity among the different groups [56]. All measures were bootstrapped (n = 10,000, indicated by the letter “*b*”) to compare groups with different sample sizes. A small sample size correction (indicated by the subscript letter “*c*”) was applied to increase the accuracy of the comparisons, enabling the comparison of niches of groups with different sample sizes [55].

## Results

### Differences in the isotopic signatures of resources and consumers among land uses

**Fig 2:**
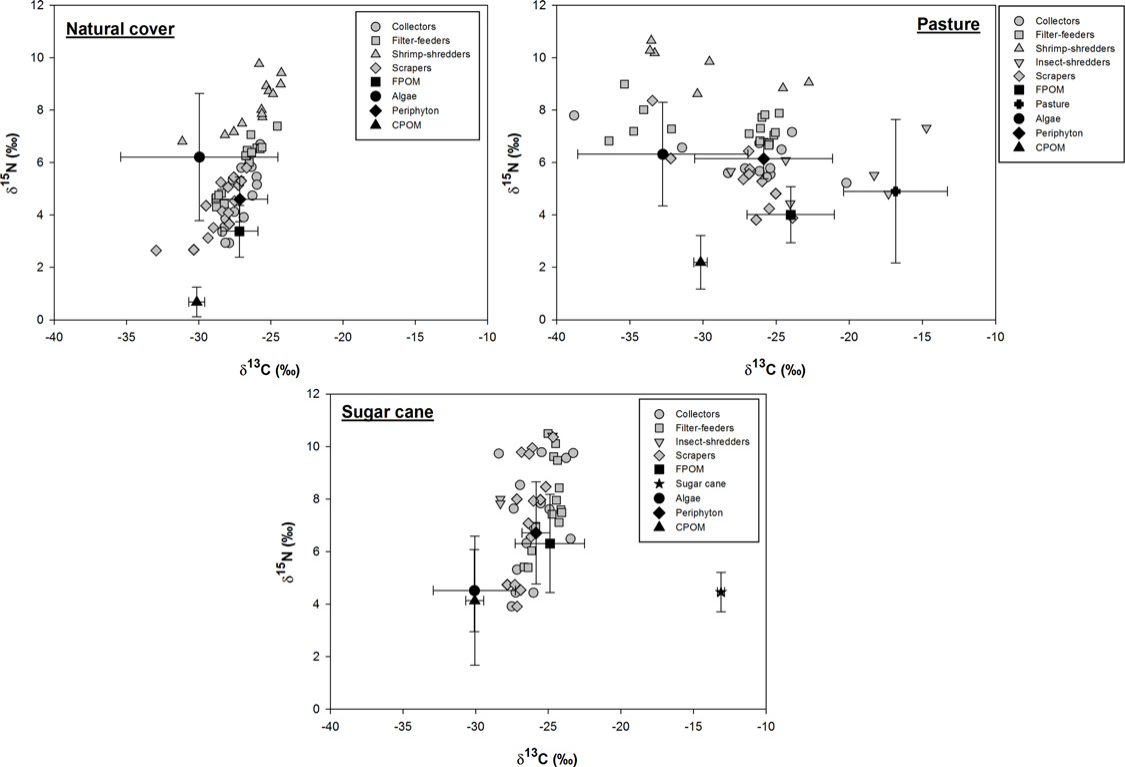
Representation of the *δ*^13^C and *δ*^15^N of food resources (mean ± SD) and consumers in sites with different riparian land uses.

The isotopic signatures of the food resources studied varied widely among and within land use types (Fig 2). In the natural cover sites, the periphyton and FPOM exhibited the highest *δ*^13^C values, whereas CPOM had the lowest values. The pasture and sugar cane plants exhibited the highest *δ*^13^C values, whereas CPOM and algae had the lowest *δ*^13^C values (Fig 2). CPOM had the lowest *δ*^15^N values in all land uses, algae exhibited the highest *δ*^15^N values in the natural cover and pasture sites, and periphyton had the highest *δ*^15^N values in sugar cane sites (Fig 2).

**Fig 3:**
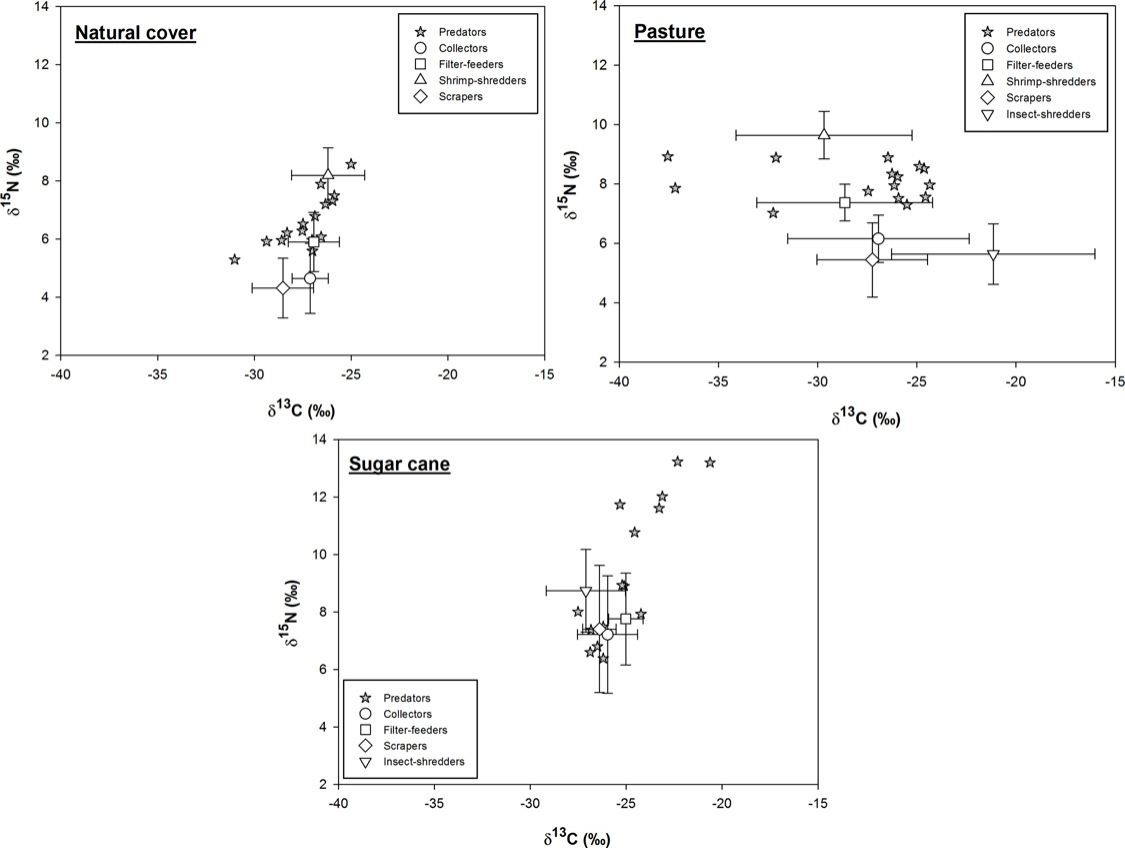
Representation of the *δ*^13^C and *δ*^15^N of prey (mean ± SD) and predators in sites with different riparian land uses.

The consumers also exhibited wide variations in isotopic composition among land uses and functional feeding groups (Figs 2 and 3). In the natural cover sites, shrimp-shredders had the highest *δ*^13^C and *δ*^15^N values, whereas scrapers had the lowest *δ*^13^C and *δ*^15^N values. In pasture sites, insect-shredders had the highest *δ*^13^C values, whereas shrimp-shredders and filter-feeders had the lowest *δ*^13^C values. Also shrimp-shredders exhibited the highest *δ*^15^N values, whereas scrapers and insect-shredders had the lowest *δ*^15^N values (Figs 2 and 3). In sugar cane sites, predators (on average) exhibited the highest *δ*^13^C and *δ*^15^N values (Fig 3, S2 Table), whereas the lowest *δ*^13^C values were recorded for insect-shredders, and the lowest *δ*^15^N values were recorded for collectors and scrapers (Fig 3).

### Feeding contribution in each stream category

**Fig 4:**
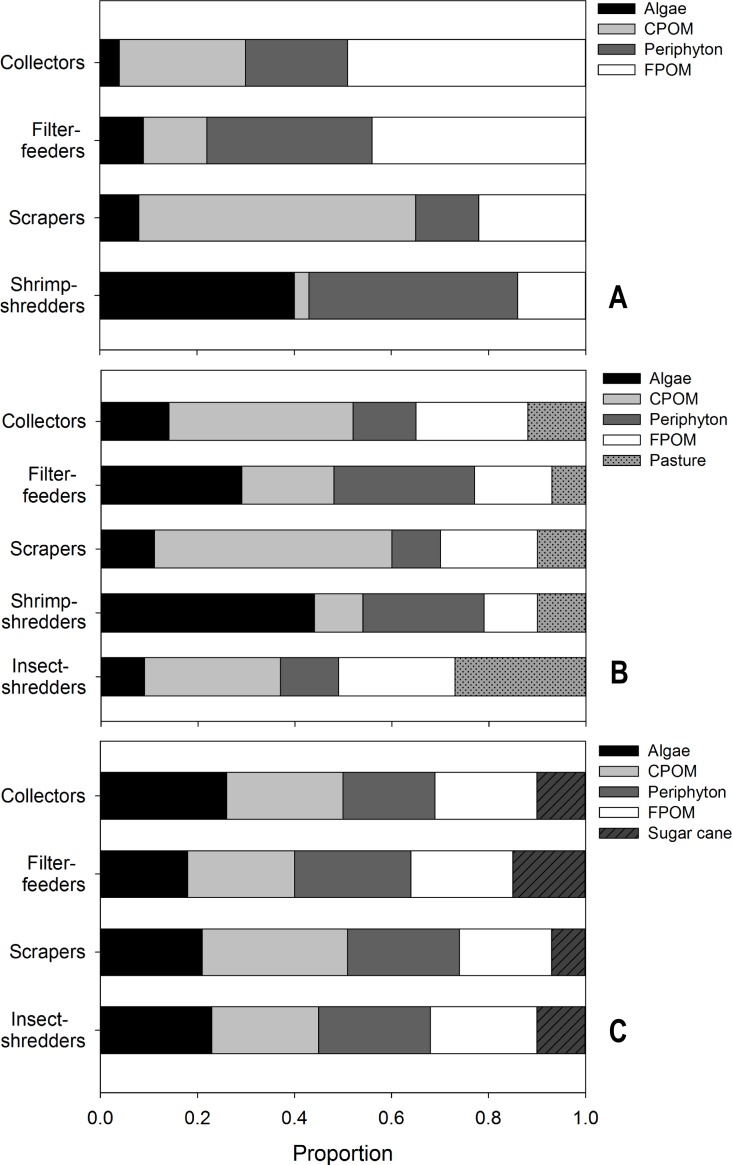
Means of the proportions of food resources used by each trophic group in each land use category based on stable isotopes analysis in R (SIAR) output: (A) natural cover, (B) pasture, and (C) sugar cane.

There was wide variation in the proportion of items assimilated by the functional feeding groups among the three land uses assessed. Sites with natural vegetation cover supported macroinvertebrate assemblages with more specialist trophic habits. Collectors and filter-feeders assimilated 44–49% FPOM, scrapers assimilated 57% CPOM, and shrimp-shredders assimilated 83% algae and periphyton as opposed to CPOM (Fig 4A, S3 Table).

Some functional groups showed greater specificity for certain food resources in pasture streams. Collectors and scrapers assimilated 38–49% CPOM, whereas the filter-feeders assimilated 58% algae and periphyton. The insect-shredders assimilated nearly equal amounts of CPOM, FPOM, and pasture grasses. However, the shrimp-shredders continued assimilating mainly algae and periphyton (Fig 4B, S3 Table).

All functional feeding groups had more generalist trophic habits in the sugar cane sites, and none assimilated a single resource in particular. In addition, the sugar cane leaves contributed only 7–15% to the diets of macroinvertebrates (Fig 4C, S3 Table).

**Fig 5:**
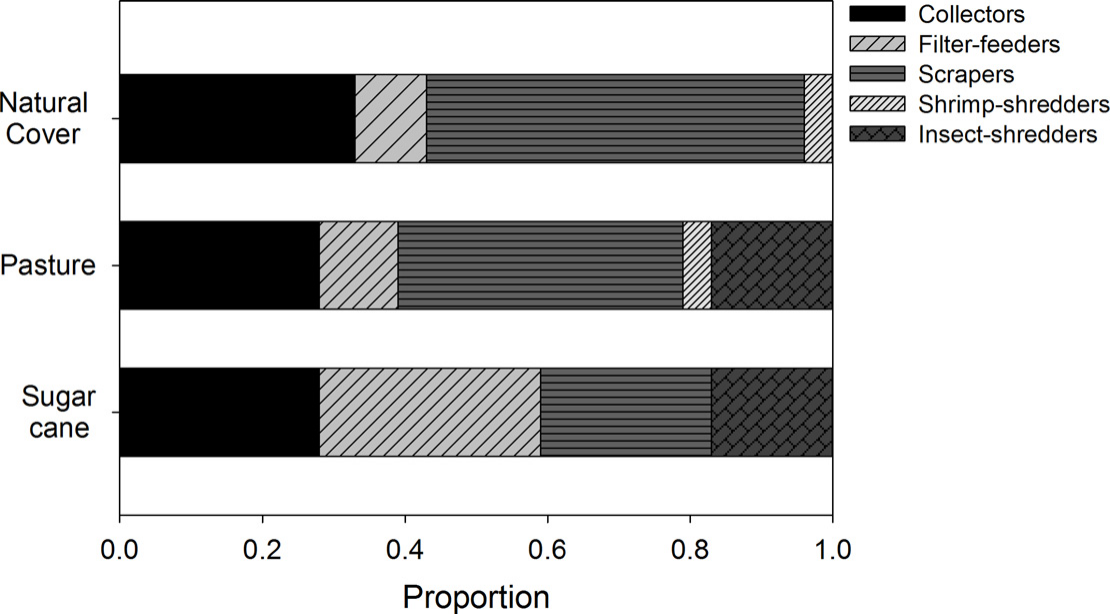
Means of the proportions of macroinvertebrate prey consumed by predators in each land use category based on stable isotopes analysis in R (SIAR) output.

Predators had a pattern similar to that of the other trophic groups in the sugar cane sites, with no preferentially assimilated resource. Predators assimilated 40% and 53% scrapers in pasture sites and sites with natural cover, respectively (Fig 5, S4 Table).

### Spatial differences in trophic structure

**Fig 6:**
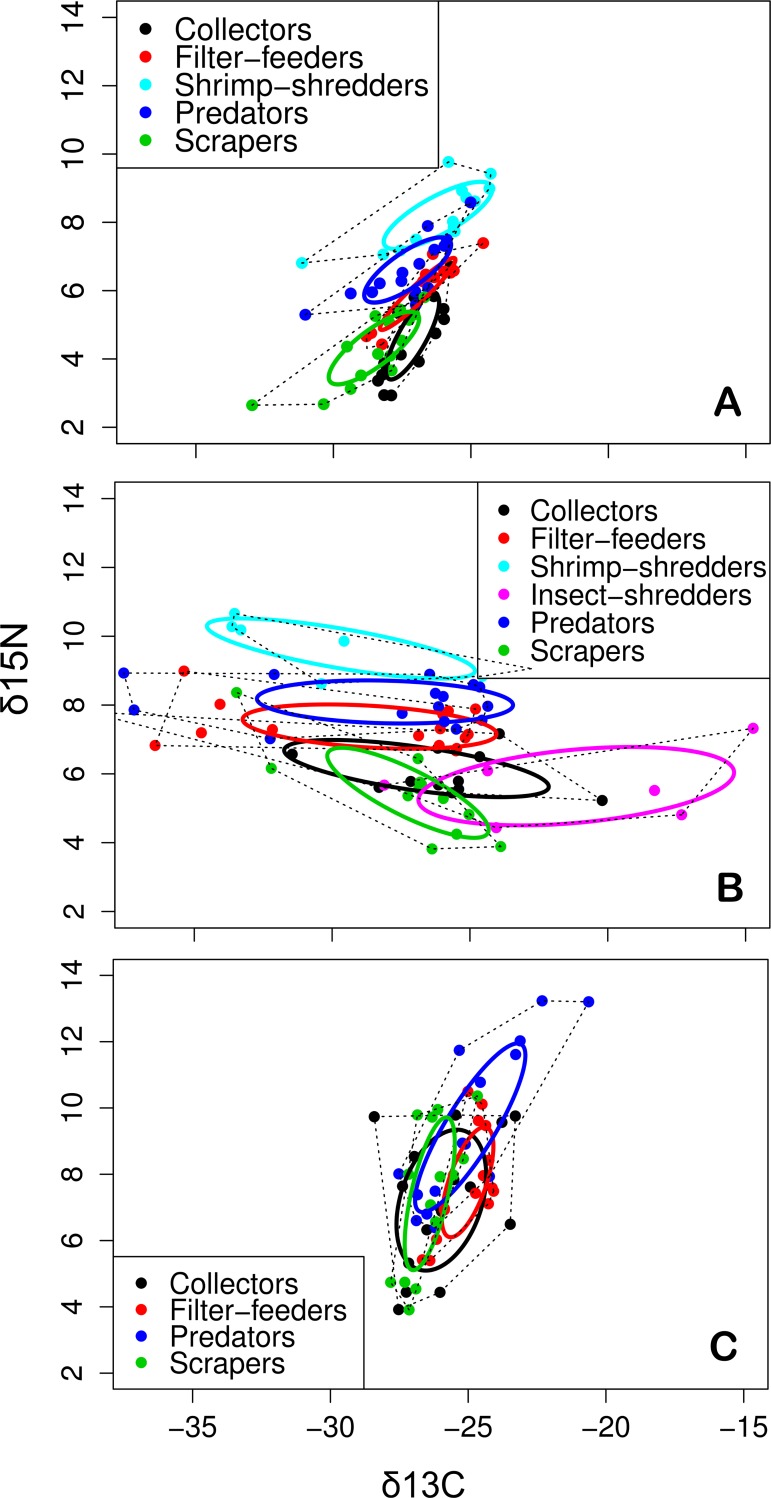
**Standard ellipse areas (SEA, solid lines), representing the core isotopic niche space of the macroinvertebrate feeding groups, as determined through SIBER models for the three land use categories: (A) natural cover, (B) pasture and (C) sugar cane.** The dashed lines delimit the total area of the macroinvertebrate assemblages of each land use category.

The standard ellipses (SEA_c_) based on the isotope ratio of trophic groups of macroinvertebrates differed in size, shape and position in the *δ*^13^C vs *δ*^15^N bi-plot space (Fig 6). The groups with the lowest SEA_c_ values occurred in the sites with natural vegetation, followed by the sugar cane and pasture sites, which exhibited the highest SEA_c_ values.

Sites with natural riparian vegetation cover exhibited very little overlap of isotopic niches (i.e., overlap of standard ellipse areas) of the macroinvertebrate feeding groups (Fig 6A). Small overlaps were observed between the collectors, scrapers and filter-feeders. The shrimp-shredders exhibited the largest SEA_c_ (3.83‰^2^), followed by scrapers (3.29‰^2^), predators (3.01‰^2^), collectors (2.26‰^2^) and filter-feeders (1.42‰^2^).

Considerable niche overlapping was observed among feeding groups in pasture sites (Fig 6B), especially between collectors and insect-shredders (30.7%) and scrapers (29.3%), and between filter-feeders and predators (25.4%). The other groups exhibited little or no niche overlap. The greatest SEA_c_ values were observed for the insect-shredders (19.2‰^2^), followed by the collectors (9.67‰^2^), predators (9.04‰^2^), shrimp-shredders (8.89‰^2^), filter-feeders (8.8‰^2^) and scrapers (6.9‰^2^).

The greatest niche overlaps among groups were observed in sugar cane sites (Fig 6C). In those sites, the niches of all feeding groups evaluated overlapped, with the highest overlap values observed between collectors and scrapers (45.9%) and collectors and filter-feeders (31.4%). The niches of predators overlapped with the niches of all other groups, whereas the filter-feeders and scrapers exhibited the least niche overlap (24.7%). The collectors had the largest SEA_c_ (10.19‰^2^), followed by predators (8.23‰^2^), scrapers (4.96‰^2^) and filter-feeders (3.71‰^2^). Calculation of the SEA_c_ for insect-shredders in sugar cane sites was not possible because we obtained only three samples of this group.

The trophic niche metrics of the macroinvertebrate communities varied among land uses. Pasture sites had significantly greater ranges of resources exploited (NR_b_ and CR_b_), trophic diversity (CD_b_), and trophic redundancy (MNND_b_) and showed significantly lower group uniformity (SDNND_b_) (Table 2). In contrast, sugar cane sites exhibited the lowest values for all metrics and sites with natural cover had intermediate values that did not overlap with sugar cane sites except for SDNND_b_ (Table 2).

**Table 2:** Layman stable isotope metrics (mean and 95% credible intervals) for each land use category: NR_b_ = *δ*^15^N range; CR_b_ = *δ*^13^C range; CD_b_ = mean distance to centroid; MNND_b_ = mean nearest neighbor distance; and SDNND_b_ = standard deviation of mean distance to centroid.

Land use	NR_b_	CR_b_	CD_b_	MNND_b_	SDNND_b_
Natural cover	3.93 (3.68–4.19)	2.39 (1.96–2.79)	1.42 (1.33–1.53)	1.28 (1.15–1.40)	0.54 (0.37–0.69)
Pasture	4.35 (4.01–4.67)	9.05 (7.47–10.5)	2.79 (2.46–3.09)	2.21 (1.92–2.49)	1.75 (1.16–2.25)
Sugar cane	2.45 (0.89–2.95)	1.70 (1.41–1.97)	1.08 (0.90–1.25)	1.07 (0.89–1.25)	0.52 (0.26–0.73)

## Discussion

The use of stable isotopes along with analytical techniques, such as the Bayesian approach, allowed (1) identification of the main resources consumed by benthic macroinvertebrates and (2) assessment of how the different land uses affected resource availability and trophic dynamics in tropical streams. Our hypotheses were corroborated because we observed macroinvertebrate assemblages with wider trophic niches and greater niche overlap in altered sites and greater resource specialization in sites with natural vegetation.

All trophic groups and virtually all resources evaluated had higher *δ*^15^N values in sugar cane and pasture sites. Nutrients from agriculture and cattle strongly affect the waterbodies and may have been responsible for the high *δ*^15^N values found in those streams. On sugar cane plantations, vinasse, a byproduct of ethanol distillation, is the main fertilizer used, whereas chemical fertilizers and livestock manure are the main sources of residues and nutrients in pastures [27]. Fertilizers increase nitrification, leading to soil ^15^N enrichment [57]. Agricultural residues usually have high *δ*^15^N ratios [58], and most are carried into waterbodies and incorporated into food webs, thus changing the *δ*^15^N available in food resources and consumers [59,60].

Although we categorized the groups *a priori*, macroinvertebrates had more generalist feeding habits at pasture and sugar cane sites, whereas more specialization occurred in macroinvertebrate assemblages of sites with natural riparian vegetation. Benstead & Pringle [61] reported similar results in a comparison of sites with preserved and deforested vegetation in Madagascar. They observed a simplification of the aquatic macroinvertebrate assemblages associated with a loss of specialist taxa associated with changes in the relative importance of the basal food resources. Therefore, we believe that land use changes lead to the selection of more generalist organisms and the elimination of more specialized organisms.

We found some unexpected results in our study; for example, scrapers assimilated more CPOM than periphyton, a result found in pasture and natural vegetation sites. According to Marchese et al. [62], who observed a high contribution of CPOM to chironomids and oligochaetes, this resource is highly colonized by bacteria, protozoa and algae, which may explain the preference for it. However, it is important to highlight that the classification of organisms into functional feeding groups is primarily related to morphology, feeding habits, or food acquisition and not to the food type per se [14]. Future studies should assess whether macroinvertebrate groups/guilds with wider trophic niches contain more generalists consuming a wide variety of food types or whether the organisms specialize in a different but narrower ranges of food resources [28].

In contrast with observations made for some fish species in the same region [27] and in other sites [10], in which was observed assimilation of sugar cane and grasses, these food resources were barely assimilated by the trophic groups evaluated. Although the FPOM was slightly richer in ^13^C in pasture and sugar cane sites, its contribution to the trophic chain was very low. The C4 plants (grasses in general, such as pasture and sugar cane) are considered to have low nutritional quality compared with C3 plants and are little used by aquatic consumers in many cases, either because of their physical or chemical characteristics that reduce consumption or because consumers are able to select other higher-quality resources [63]. Although present in large amounts at the pasture and sugar cane sites, little C4 plant material entered the food chain through aquatic macroinvertebrates. Bunn et al. [64] and Martinelli et al. [65] also found that few C4 resources were incorporated into the trophic chain in anthropogenically altered sites, despite representing >50% of the detritus in the systems. This reduced contribution of C4 resources into aquatic trophic chains shows how the conversion of natural riparian vegetation into sugar cane plantations and pastures has the potential to alter the trophic dynamics and functional organization of aquatic communities, leading to substantial changes in stream ecosystem functions.

Pasture sites had autochthonous resources (algae and periphyton) with the widest *δ*^13^C and *δ*^15^N ranges. This wider range may result from reduced riparian canopy cover and, consequently, higher light input, promoting higher diversity and abundance of algae and periphyton species. Similar results have been reported by Turner & Edwards [66], where the producers (algae) had more widely dispersed *δ*^13^C and *δ*^15^N values. They argued that this variation might result from greater taxonomic diversity of the producers, which could lead to greater diversity in C and N metabolism.

Consumers from streams with natural riparian vegetation had the narrowest isotopic niches and the lowest niche overlaps. This pattern indicates that the macroinvertebrate functional groups in those sites had more selective feeding habits with a lower overlap of trophic niches and, consequently, less competition for resources. In their natural state, wooded riparian zones are effective in preserving the ecological integrity and trophic dynamics of aquatic ecosystems. Land use changes and the consequent shifts in the input of allochthonous nutrients and autochthonous production can reduce the balance between functional feeding groups [67] and, widen their trophic niches.

The isotopic metrics calculated for the assemblages described in this study were based on the mean *δ*^13^C and *δ*^15^N values of multiple individuals for each group in a trophic web, and such intraspecific variation was not considered in this analysis (i.e. species within the same FFG) [21]. The highest mean dNR_b_ and dCR_b_ values observed in the pasture sites indicate that macroinvertebrates use a wider range of the available food sources in this environment, especially in contrast with the sugar cane sites. This result is consistent with the SEA_c_ values, which were highest in pasture sites. The highest mean CD_b_ value was also observed in pasture sites, indicating higher trophic diversity at those sites. The highest mean MNND_b_ values were observed for pasture sites, indicating low trophic redundancy. However, the highest SDNND_b_ values were also observed in the pasture sites, suggesting the existence of a less uniform trophic niche distribution, despite the low trophic redundancy. This is most likely a result of the greater availability of periphyton and algae in this environment, which are resources with wider *δ*^13^C ranges and which are used by many consumers. According to Layman et al. [21], MNND_b_ and SDNND_b_ increase because consumers have more distinct trophic positions (greater distance among consumers in isotopic space).

Another noteworthy result is the occurrence of the crustacean, *Macrobrachium amazonicus*, considered a shrimp-shredder in this study, which is an alien species in the region found in pasture and in natural cover sites. The isotopic niche (SEA_c_) of shrimp-shredders was one of the highest compared with the other functional groups in those land uses. However, the niche of shrimp-shredders did not overlap with the others, suggesting that this group is exploiting resources that would otherwise not be fully exploited by the native fauna. In addition, the shrimp-shredders were the group least preyed upon, with virtually no contribution to predators. Recently in a global evaluation of the consequences of non-native species on the isotopic structure of freshwater fish communities, Sagouis et al. [68] found that communities in lotic ecosystems containing non-native species had a larger total isotopic niche than communities without non-native species and those non-native species were mainly located at the edges of the isotopic niche. Thus, we highlight the importance of studies assessing how invasive alien species are directly competing for resources with native species or not (e.g., [56,69,70]), where the comparison of isotopic niches and assimilated items may be important tools.

We conclude that land use changes, such as sugar cane culture and livestock pasturing, may lead to benthic macroinvertebrate assemblages with more generalist feeding behaviors and higher trophic niche overlap. In addition, our results reinforce the idea that stable isotope analysis is a relevant tool for biomonitoring and evaluating the effects of land use changes on the dynamics and functioning of tropical streams.

## Supporting Information

S1 Table Physical characteristics, land use and environmental variables calculated to the nine streams in the three land use categories. The numbers 1, 2 and 3 correspond to each of the three streams sampled in each land use category (See Fig 1). Order = rank of the stream orders according to Strahler; Veg. cover = Vegetation Cover. All environmental variables were calculated according to the proportion in which they occur in each assessed stream.
(DOCX)

S2 Table Mean ± S.D. isotopic signatures of resources and consumers sampled in the three land use categories. The letters *a* and *b* indicate which signatures are different according to *post hoc* test. The letter “n” indicates the number of replicates used in each group analysis.
(DOCX)

S3 Table Stable isotope analysis in R (SIAR) results of the food source proportions in the diet of the functional trophic groups (FTG) (95% confidence interval). (DOCX)

S4 Table Stable isotope analysis in R (SIAR) results of the prey proportions in predator diets (95% confidence interval). (DOCX)
