# Supplementary material for: Land Use Influences Niche Size and the Assimilation of Resources by Benthic Macroinvertebrates in Tropical Headwater Streams
Source: PLoS One. 2016 Mar 2;11(3):e0150527. doi: 10.1371/journal.pone.0150527 (PMC4774910; doi:10.1371/journal.pone.0150527)
Supplement: S1 Table — The numbers 1, 2 and 3 correspond to each of the three streams sampled in each land use category (See Fig 1). Order = rank of the stream orders according to Strahler; Veg. cover = Vegetation Cover. All environmental variables were calculated according to the proportion in which they occur in each assessed stream. (DOCX) [file pone.0150527.s001.docx]

**S1 Table: Physical characteristics, land use and environmental variables calculated to the nine streams in the three land use categories.** The numbers 1, 2 and 3 correspond to each of the three streams sampled in each land use category (See Figure 1). Order= rank of the stream orders according to Strahler; Veg. cover= Vegetation Cover. All environmental variables were calculated according to the proportion in which they occur in each assessed stream.

|  |  | **Characteristics of streams** | | | |  | **Land use** | | | |  | **Environmental variables** | | | | | |
| --- | --- | --- | --- | --- | --- | --- | --- | --- | --- | --- | --- | --- | --- | --- | --- | --- | --- |
| **Streams** | | **Altitude** | **Order** | **Mean depth** | **Mean width** |  | **Natural cover** | **Pasture** | **Cane** | **Others** |  | **Fine subst.** | **Veg. cover** | **Rapid flow** | **Aquatic plant** | **Leaf banks** | **Algae** |
|  |  | **(m)** |  | **(m)** | **(m)** |  | **(%)** | **(%)** | **(%)** | **(%)** |  | **(%)** | **(%)** | **(%)** | **(%)** | **(%)** | **(%)** |
| **Natural cover** | 1 | 482 | 2^nd^ | 0.20 | 3.42 |  | 53.14 | 0 | 46.86 | 0 |  | <0.01 | 0.94 | 0.69 | <0.01 | 0.17 | <0.01 |
|  | 2 | 434 | 3^th^ | 0.19 | 7.29 |  | 39.13 | 57.87 | 0 | 3.00 |  | <0.01 | 0.47 | 0.46 | <0.01 | 0.14 | <0.01 |
|  | 3 | 509 | 3^th^ | 0.19 | 7.35 |  | 50.86 | 49.14 | 0 | 0 |  | <0.01 | 0.90 | 0.51 | <0.01 | 0.18 | <0.01 |
| **Sugar cane** | 1 | 516 | 2^nd^ | 0.23 | 3.98 |  | 14.05 | 36.56 | 45.81 | 3.59 |  | 0.01 | 0.73 | 0.38 | <0.01 | 0.11 | <0.01 |
|  | 2 | 441 | 3^th^ | 0.19 | 1.85 |  | 14.63 | 0 | 85.37 | 0 |  | <0.01 | 0.99 | 0.17 | 0.04 | 0.16 | <0.01 |
|  | 3 | 463 | 2^nd^ | 0.27 | 1.27 |  | 0 | 43.39 | 56.61 | 0 |  | 0.03 | 1.0 | 0.67 | 0.02 | 0.07 | <0.01 |
| **Pasture** | 1 | 425 | 3^th^ | 0.64 | 3.49 |  | 8.57 | 62.35 | 0 | 29.08 |  | 0.3 | 0.33 | 0.43 | 0.66 | 0.02 | 0.22 |
|  | 2 | 461 | 3^th^ | 0.13 | 2.00 |  | 0 | 97.69 | 0 | 2.31 |  | 0.21 | 0.01 | 0.45 | 0.05 | <0.01 | 0.14 |
|  | 3 | 479 | 2^nd^ | 0.17 | 4.80 |  | 16.41 | 65.35 | 0 | 18.24 |  | 0.02 | 0.38 | 0.15 | 0.02 | 0.02 | <0.01 |
