# Supplementary material for: Land Use Influences Niche Size and the Assimilation of Resources by Benthic Macroinvertebrates in Tropical Headwater Streams
Source: PLoS One. 2016 Mar 2;11(3):e0150527. doi: 10.1371/journal.pone.0150527 (PMC4774910; doi:10.1371/journal.pone.0150527)
Supplement: S2 Table — The letters a and b indicate which signatures are different according to post hoc test. The letter “n” indicates the number of replicates used in each group analysis. (DOCX) [file pone.0150527.s002.docx]

**Table S1: Mean ± S.D. isotopic signatures of resources and consumers sampled in the three land use categories.** The letters *a* and *b* indicate which signatures are different according to *post hoc* test. The letter “n” indicates the number of replicates used in each group analysis.

|  | **δ13C** | | | | | | | | | | | | | | | |  | **δ15N** | | | | | | | | | | | | | | | |
| --- | --- | --- | --- | --- | --- | --- | --- | --- | --- | --- | --- | --- | --- | --- | --- | --- | --- | --- | --- | --- | --- | --- | --- | --- | --- | --- | --- | --- | --- | --- | --- | --- | --- |
| **Resources** | Natural cover | | |  | n | Pasture | | |  | n | Sugar cane | | |  | n | p |  | Natural cover | | |  | n | Pasture | | |  | n | Sugar cane | | |  | n | p |
| Algae | -29.96 | ± | 5.44 |  | 10 | -32.76 | ± | 5.81 |  | 10 | -30.10 | ± | 2.83 |  | 5 | 0.45 |  | 6.21 | ± | 2.43 |  | 10 | 6.32 | ± | 1.98 |  | 10 | 4.52 | ± | 1.56 |  | 5 | 0.27 |
| CPOM | -30.15 | ± | 0.55 |  | 30 | -30.17 | ± | 0.46 |  | 17 | -30.08 | ± | 0.61 |  | 30 | 0.91 |  | 0.68 | ± | 0.56 | *b* | 30 | 2.19 | ± | 1.03 | b | 17 | 4.13 | ± | 2.46 | *a* | 30 | **<0.01** |
| Grasses | − |  | − |  | − | -16.83 | ± | 3.55 |  | 15 | − |  | − |  | − | − |  | − |  | − |  | − | 4.90 | ± | 2.74 |  | 15 | − |  | − |  | − | − |
| Periphyton | -27.16 | ± | 1.93 |  | 15 | -25.86 | ± | 4.71 |  | 15 | -25.84 | ± | 0.97 |  | 15 | 0.39 |  | 4.60 | ± | 0.85 | *b* | 15 | 6.15 | ± | 0.77 | a | 15 | 6.71 | ± | 1.94 | *a* | 15 | **<0.01** |
| Sugarcane | − |  | − |  | − | − |  | − |  | − | -13.11 | ± | 0.25 |  | 10 | − |  | − |  | − |  | − | − |  | − |  | − | 4.46 | ± | 0.75 |  | 10 | − |
| FPOM | -27.18 | ± | 1.28 | *b* | *17* | -24.01 | ± | 2.98 | *a* | *18* | -24.88 | ± | 2.39 | *a* | *25* | **<0.01** |  | 3.38 | ± | 0.98 | *b* | *17* | 4.01 | ± | 1.07 | b | *18* | 6.31 | ± | 1.87 | *a* | *25* | **<0.01** |
| **Consumers** |  |  |  |  |  |  |  |  |  |  |  |  |  |  |  |  |  |  |  |  |  |  |  |  |  |  |  |  |  |  |  |  |  |
| Collector | -27.12 | ± | 0.93 |  | 15 | -26.94 | ± | 4.58 |  | 12 | -25.97 | ± | 1.57 |  | 15 | 0.10 |  | 4.65 | ± | 1.20 | *b* | *15* | 6.15 | ± | 0.79 | a | 12 | 7.22 | ± | 2.05 | *a* | 15 | **<0.01** |
| Filter | -26.94 | ± | 1.32 | *a* | 15 | -28.64 | ± | 4.44 | *a* | 15 | -25.02 | ± | 0.90 | *b* | 15 | **<0.01** |  | 5.90 | ± | 1.01 | *b* | *15* | 7.37 | ± | 0.62 | a | 15 | 7.76 | ± | 1.60 | *a* | 15 | **<0.01** |
| Shrimp-shredder | -26.20 | ± | 1.89 |  | 13 | -29.68 | ± | 4.44 |  | 7 | − |  | − |  | − | 0.12 |  | 8.20 | ± | 0.94 | *b* | *13* | 9.64 | ± | 0.80 | a | 7 | − |  | − |  | − | **<0.01** |
| Insect-shredder | − |  | − |  | − | -21.14 | ± | 5.12 |  | 6 | -27.10 | ± | 2.07 |  | 3 | 0.10 |  | − |  | − |  | − | 5.64 | ± | 1.02 | b | 6 | 8.75 | ± | 1.44 | *a* | 3 | **<0.05** |
| Predator | -27.30 | ± | 1.52 | *b* | 15 | -28.08 | ± | 4.48 | *ab* | 15 | -24.93 | ± | 1.93 | *a* | 15 | **<0.01** |  | 6.61 | ± | 0.92 | *b* | *15* | 8.09 | ± | 0.60 | a | 15 | 9.40 | ± | 2.45 | *a* | 15 | **<0.01** |
| Scraper | -28.53 | ± | 1.58 | *b* | 15 | -27.25 | ± | 2.79 | *a* | 12 | -26.39 | ± | 0.87 | *a* | 14 | **<0.01** |  | 4.32 | ± | 1.03 | *c* | *15* | 5.45 | ± | 1.25 | b | 12 | 7.41 | ± | 2.22 | *a* | 14 | **<0.01** |
