# Supplementary material for: Land Use Influences Niche Size and the Assimilation of Resources by Benthic Macroinvertebrates in Tropical Headwater Streams
Source: PLoS One. 2016 Mar 2;11(3):e0150527. doi: 10.1371/journal.pone.0150527 (PMC4774910; doi:10.1371/journal.pone.0150527)
Supplement: S3 Table — (DOCX) [file pone.0150527.s003.docx]

**Table S2. Stable isotope analysis in R (SIAR) results of the food source proportions in the diet of the functional trophic groups (FTG) (95% confidence interval).**

|  |  | **Consumers** | | | | |
| --- | --- | --- | --- | --- | --- | --- |
| **Category** | **Resources** | Collectors | Filter-feeders | Shrimp-shredders | Insect-shredders | Scrapers |
| Natural Cover | Algae | 0.04 (0.00-0.10) | 0.09 (0.00-0.22) | 0.40 (0.14-0.60) | ­- | 0.08 (0.00-0.19) |
|  | CPOM | 0.26 (0.11-0.43) | 0.13 (0.00-0.26) | 0.03 (0.00-0.09) | ­- | 0.57 (0.40-0.72) |
|  | Periphyton | 0.21 (0.00-0.43) | 0.34 (0.02-0.61) | 0.43 (0.11-0.74) | ­- | 0.13 (0.00-0.31) |
|  | FPOM | 0.49 (0.19-0.78) | 0.44 (0.11-0.81) | 0.14 (0.00-0.36) | ­- | 0.22 (0.00-0.44) |
| Pasture | Algae | 0.14 (0.00-0.29) | 0.29 (0.13-0.45) | 0.44 (0.16-0.76) | 0.09 (0.00-0.25) | 0.11 (0.00-0.27) |
|  | CPOM | 0.38 (0.21-0.54) | 0.19 (0.05-0.32) | 0.10 (0.00-0.28) | 0.28 (0.02-0.49) | 0.49 (0.26-0.72) |
|  | Grasses | 0.12 (0.00-0.26) | 0.07 (0.00-0.18) | 0.10 (0.00-0.26) | 0.27 (0.01-0.48) | 0.10 (0.00-0.22) |
|  | Periphyton | 0.13 (0.00-0.30) | 0.29 (0.08-0.50) | 0.25 (0.00-0.48) | 0.12 (0.00-0.30) | 0.10 (0.00-0.27) |
|  | FPOM | 0.23 (0.00-0.44) | 0.16 (0.00-0.34) | 0.11 (0.00-0.30) | 0.24 (0.00-0.45) | 0.20 (0.00-0.41) |
| Sugar cane | Algae | 0.26 (0.01-0.47) | 0.18 (0.00-0.33) | ­- | 0.23 (0.00-0.43) | 0.21 (0.01-0.37) |
|  | CPOM | 0.24 (0.01-0.44) | 0.22 (0.02-0.39) | ­- | 0.22 (0.00-0.42) | 0.3 (0.09-0.50) |
|  | Periphyton | 0.19 (0.00-0.38) | 0.24 (0.01-0.44) | ­- | 0.23 (0.00-0.44) | 0.23 (0.01-0.43) |
|  | Sugarcane | 0.10 (0.02-0.19) | 0.15 (0.07-0.21) | ­- | 0.10 (0.00-0.27) | 0.07 (0.00-0.14) |
|  | FPOM | 0.21 (0.00-0.41) | 0.21 (0.01-0.38) | ­- | 0.22 (0.00-0.42) | 0.19 (0.00-0.37) |
