# Supplementary material for: Land Use Influences Niche Size and the Assimilation of Resources by Benthic Macroinvertebrates in Tropical Headwater Streams
Source: PLoS One. 2016 Mar 2;11(3):e0150527. doi: 10.1371/journal.pone.0150527 (PMC4774910; doi:10.1371/journal.pone.0150527)
Supplement: S4 Table — (DOCX) [file pone.0150527.s004.docx]

**Table S3: Stable isotope analysis in R (SIAR) results of the prey proportions in predator diets (95% confidence interval).**

| **Resources** | **Category** | | |
| --- | --- | --- | --- |
|  | Natural Cover | Pasture | Sugar cane |
| Collectors | 0.33 (0.00-0.61) | 0.28 (0.01-0.54) | 0.28 (0.00-0.54) |
| Filter-feeders | 0.10 (0.00-0.29) | 0.11 (0.00-0.26) | 0.31 (0.01-0.57) |
| Shrimp-shredders | 0.04 (0.00-0.11) | 0.04 (0.00-0.10) | ­- |
| Insect-shredders | ­- | 0.17 (0.00-0.37) | 0.17 (0.00-0.38) |
| Scrapers | 0.53 (0.24-0.85) | 0.40 (0.17-0.64) | 0.24 (0.00-0.48) |
